# Stock Assessment of *Nemipterus marginatus* in Java Sea: Case Study of Batang Regency and Pati Regency

**DOI:** 10.21315/tlsr2026.37.3.4

**Published:** 2026-10-15

**Authors:** Dian Wijayanto, Faik Kurohman, Hendrik A. Setyawan, Dita J. Kurnia

**Affiliations:** Faculty of Fisheries and Marine Science, Universitas Diponegoro, Tembalang, Semarang City, Central Java 50275, Indonesia

**Keywords:** Central Java, Exploitation Rate, Maximum Sustainable Yield, MSY, *Nemipterus marginatus*

## Abstract

This research examined the expolitation status of *Nemipterus marginatus* in the coastal waters of Pati and Batang Regencies, Central Java Province. Field surveys were conducted from April to September 2025 to measure the total length and body weight of 226 specimens collected from Pati and 391 specimens from Batang. The length–weight relationship of *N. marginatus* in Pati and Batang show negative allometric pattern, with *W =* 0.134L^2.15^ and *W =* 0.052L^2.471^, respectively. The *Lc*_50_*_%_* values were 11.61 cm in Pati and 10.90 cm in Batang. Growth parameters for *N. marginatus* in Pati were *L*∞ = 26.0 cm, *K* = 0.40 year^−1^, *M* = 1.02 year^−1^, *F* = 0.76 year^−1^, *Z* = 1.78 year^−1^ and *E* = 0.43, indicating a sustainable level of exploitation. Meanwhile, in Batang, *L*∞ *=* 26.0 cm, *K =* 0.58 year^−1^, *M* = 1.30 year^−1^, *F* = 1.40 year^−1^, *Z* = 2.70 year^−1^ and *E* = 0.52 (overexploitation). The estimated Maximum Sustainable Yield (MSY) was 3.286 tons (IDR29.574 million) in Pati and 3.589 tons (IDR32.302 million) in Batang. The recruitment season was estimated to occur from February to July in Pati and from February to June, with secondary peaks in September and November, in Batang. These findings indicate that *N. marginatus* populations in the northern waters of Central Java are approaching optimal exploitation levels. Hence, management strategies should consider the size limits and seasonal fishing regulations to promote stock sustainability.

**HIGHLIGHTS**

- Different exploitation status between Pati and Batang: In Pati, the exploitation rate (E = 0.43) indicates a sustainable condition and inBatang, the exploitation rate (E = 0.52) indicates overexploitation.This shows that fishing pressure is significantly higher in Batangwaters.
- Most fish are caught before reaching sexual maturity: The lengthat first capture (Lc_50_%) (10.90–11.61 cm) is smaller than the length at first maturity (Lm_50_%) (~13–14 cm). This means that most individuals are harvested before they can reproduce.
- Stock is approaching optimal exploitation → management is needed: Production in Batang has exceeded MSY, while Pati remains below it. Recruitment occurs seasonally (main peak: February–July).
- Recommended management strategies include minimum catch size regulation, seasonal fishing restrictions and control of fishing effort.

## INTRODUCTION

*Nemipterus marginatus* (common name: red filament threadfin bream; local names: *kurisi*, *kurisi merah*, *kuniran*) is a demersal marine fish species of family Nemipteridae (Paul *et al*. 2018; Al-Buwaiqi *et al*. 2019; Ismaili *et al*. 2023; Gao *et al*. 2024; Murugesan *et al*. 2025). This species inhabits sandy or muddy substrates at depths ranging from shallow coastal waters to approximately 100 m, including in Indo-West Pacific region (El-Ganainy *et al*. 2018; Froese & Pauly 2019; 2025; *Thao et al*. 2022). The school of *N. marginatus* is frequently captured using bottom fishing gears such as trawls, gillnets and other demersal gear types (Pauly & Martosubroto 1980; Russell 1990; Ning *et al*. 2011; *He* et al. 2021).

*N. marginatus* is carnivore that feed on small crustaceans (including shrimps), polychaetes, small mollusks and benthic fishes (Sululu *et al*. 2017; Tonnie *et al*. 2018; Paul *et al*. 2018; Vaisakh *et al*. 2019; Detcharoen *et al*. 2024). Its spatial distribution and population density are influenced by the availability of benthic prey and environmental parameters such as temperature, salinity and local hydrodynamics (Shafeeque *et al*. 2021; Ritonga *et al*. 2022; Mahmoud & Ibrahim 2024). The species migrate within local area for feeding, spawning or responding to environmental fluctuations (El-SiKaily 2024).

The use of demersal fisheries using trawls, gillnets and other bottom gears to capture *N. marginatus* puts the species on the risk of overexploitation (Hong *et al*. 2024). Although often regarded as a secondary catch, the widespread use of non-selective fishing methods poses risks of excessive bycatch and potential overfishing, especially in areas lacking effective stock management (Gilman *et al*. 2019; Schwermer *et al*. 2020; Wibisono *et al*. 2022). *N. marginatus* maintains the benthic food web dynamics within the ecosystem (Ahmad *et al*. 2015; Grgec *et al*. 2020). Prior researchers reported heavy exploitation of the fisheries in Java Sea which is closer to the critical thresholds (Dimarchopoulou *et al*. 2023; Nugroho *et al*. 2025). Unselective fishing practices may alter benthic community structures and reduce biodiversity (Ren & Liu 2020; Herwaty *et al*. 2023) that are detrimental to species with low recovery potential (Williams *et al*. 2013; Ernawati *et al*. 2024).

The use traditional demersal gears in Indonesia, such as *cantrang* (Danish trawl), has intensified fishing pressure on bottom-dwelling species (Taurusman *et al*. 2025). Unfortunately, the fishery resources at the northern coast of Central Java, including Pati and Batang Regencies are not sufficiently evaluated, thereby requiring re-evaluation. The potential influence of fishing intensity and environmental changes (e.g., rising sea temperatures, sedimentation and habitat degradation) on stock structure and long-term sustainability has been discussed in general fisheries literature (Goethel *et al*. 2019; Peltonen & Weigel 2022), although the direct relevance of these parameters to *N. marginatus* remains unclear due to limited species-specific evidence. This study aims to examine the current exploitation status and ecological condition of *N. marginatus* in the northern coastal waters of Central Java, particularly in Pati and Batang, as a basis for sustainable demersal fisheries management.

## MATERIALS AND METHODS

### Research Time and Location

This study was conducted in the coastal waters of Pati and Batang Regencies, Central Java Province, Indonesia (Fig. 1). Fish sampling and field data collection were carried out from April to September 2025 at major fish landing sites in both regencies.

### Data Collection and Analysis

Several biological parameters were observed, including body weight (g) and total length (TL, in cm) of *N. marginatus* (see Fig. 2). A total of 226 *N. marginatus* specimens were randomly selected from Pati Regency and 391 samples were collected from Batang.

Data analysis was performed using Microsoft Excel and FiSAT II software. The growth function was modeled using the von Bertalanffy’s growth equation, while the L∞ and K values were measured using the ELEFAN I (Electronic Length Frequency Analysis) method in FiSAT II. The t_0_ value was estimated using the logarithmic equation of Gulland (1983). Total mortality (*Z*) was calculated through the length-converted catch curve module in FiSAT II. Natural mortality (*M*) was estimated using Pauly’s empirical equation. Fishing mortality (F) was then measured based on the gap between *Z* and *M* (*F = Z* − *M*). The exploitation ratio or exploitation rate (*E*), length-weight relationship (allometric model) and length at first capture (Lc_50%_) were also examined (Gulland 1965; Sparre & Venema 1998; FAO 1998; Gayanilo *et al*. 2005; Badrudin *et al*. 2011; Kindong *et al*. 2018; Wang *et al*. 2020; Sarr *et al*. 2023; Tirtadanu *et al*. 2023; Bhakta *et al*. 2024; Wijayanto *et al*. 2025; Riza *et al*. 2026). The formulas used in the analysis are as follows:


(1) $$W=a\;L^{b}$$

(2) Ln W=Ln a+b Ln CL

(3) $$Lt=L\infty \;(1-e^{-k(t-t_{o})})$$

(4) $$Log(-t_{0})=-0.3922-0.2752\;Log\;L\infty -1.038\;Log\;K$$

(5) $$\begin{array}{ll} Log(M)=\; & -0.0066-0.279\;Log\;L\infty \\ & +0.6543\;Log\;K\\ & +0.4634\;Log\;T \end{array}$$

(6) F=Z-M

(7) $$E=\frac{F}{Z}$$

(8) $$E_{MSY}=0.5$$

(9) $$F_{MSY}=0.5\;\text{Z}$$

(10) $$C_{MSY}=\frac{F_{MSY}}{F}C$$

Where *W* = fish weight (g); *Lt* = total length at age *t* (cm); *a* and *b* = length-weight relationship constants; *L*_∞_ = asymptotic length (cm); *K* = growth coefficient (per year); *t* = fish age (years); *t*_0_ = theoretical age at which length equals zero (years); *T* = water temperature (assumed 28°C); *Z* = total mortality; *M* = natural mortality; *F* = fishing mortality; *E* = exploitation rate; *E_MSY_* = the optimal exploitation rate under the MSY conditions; *F_MSY_* = fishing mortality under the MSY conditions; *C_MSY_* = fish production under the MSY conditions.

## RESULTS

The analysis of the length–weight relationship of *N. marginatus* in the two study locations (Pati and Batang Regencies) is presented in Table 1. The *b* values for both locations were less than 3, namely 2.157 in Pati and 2.471 in Batang, indicating a negative allometric growth pattern, where weight increases at a slower rate than body length. The coefficients of determination (R^2^) were 70.3% for Pati and 86.5% for Batang, suggesting a positive relationship between fish length and weight in both study areas. However, the R^2^ value of 70.3% should be interpreted with caution and does not necessarily indicate a strong relationship.

The total length (TL) composition of *N. marginatus* caught by local fishers is presented in Table 3 and Fig. 3. The size distribution of fish in Pati Regency ranged from 9.01 to 21.00 cm TL, with dominant size classes found at intervals of 10.51 cm–12.00 cm (26%) and 12.01 cm–13.50 cm (16%). Meanwhile, in Batang Regency, the captured fish exhibited a wider size range of 6.00 cm–21.00 cm TL, dominated by individuals measuring 13.51 cm–15.00 cm (24%) and 9.01 cm–10.50 cm (23%). 80% of individuals in Pati and 88% in Batang had total length size under 15 cm, indicating that the fish caught were mostly juvenile fish. The size distribution pattern further confirms that the fishing activities largely targeted young individuals (Zhang *et al*. 2022b), with significantly higher proportion of small-sized fish (6 cm–10 cm TL) was recorded in Batang.

Table 2 presents the results of the Lc_50%_ analysis, in which the estimated Lc_50%_ value in Pati Regency was 11.61 cm and 10.90 cm in Batang Regency. Both values were smaller than the length at first maturity (Lm_50%_) that ranged between 13 cm–14 cm for *N. japonicus* as proposed by Lisamy *et al*. (2023). The finding shows that most *N. marginatus* individuals in both locations were caught before reaching sexual maturity, which raises the risk of overfishing (Link & Watson 2019; *Zhang et al*. 2022a).

The growth parameters of *N. marginatus* in Pati and Batang Regencies are presented in Table 4. The asymptotic length (L_∞_) values were relatively similar, at 26 cm, indicating a comparable maximum body size of the population in both locations. However, the growth coefficient (K) between sites differed, with higher value (K = 0.58 year^−1^) in Batang compared to Pati (K = 0.40 year^−1^). Both natural mortality (M) and total mortality (Z) were also higher in Batang (M = 1.30 year^−1^; Z = 2.70 year^−1^) than in Pati (M = 1.02 year^−1^; Z = 1.78 year^−1^). The fishing mortality (F = Z − M) reached 1.40 year^−1^ in Batang, resulting in an exploitation rate (E) of 0.52, indicating an over-exploited condition. Conversely, the E value of 0.43 in Pati suggests that the fishery in this area remains within sustainable limits.

The estimated Maximum Sustainable Yield (MSY) of *N. marginatus* in both sites is presented in Table 5. The actual production in Pati Regency was estimated to remain below the MSY level of 3.286 tons per year within the sustainably exploited or underexploited stock categories. The actual production in Batang Regency slightly exceeded the estimated MSY of 3.589 tons per year, indicating potential overfishing (Link & Watson 2019).

The estimated economic value of *N. marginatus* production at the MSY level reached IDR 29.574 million per year in Pati Regency and IDR 32.302 million per year in Batang Regency (market price of IDR 9,000 per kilogram). Although the economic value in Batang was higher, the fishing pressure on the stock was also greater, where long-term economic benefits may decline due to the lower stock biomass.

The recruitment season of *N. marginatus* was estimated to occur from February to July and from February to June in Pati Regency. Meanwhile, the recruitment season in Batang Regency occurs in September and November (Fig. 4). In Pati, the recruitment peaked in July (20.3%), while in Batang, the peak recruitment occurred in May (17.5%) with a secondary peak in September (12.4%). It can be inferred that the regeneration of young individuals into the stock occurs twice a year, with the main peak taking place during the first transitional season (March–July).

## DISCUSSION

This research showed a negative allometric growth pattern in *N. marginatus* as found in by previous researchers in the Java Sea and adjacent waters. Hartanti *et al*. (2017) also noticed a similar pattern for *Nemipterus* spp. in the Java Sea, with a *b* value of 2.65. Variations in *b* values may be influenced by environmental factors such as water depth, food availability and physiological conditions of the fish. Intrinsic factors (sex, fishing season and gonadal maturity stage) also affect the length-weight relationship (Ning *et al*. 2011; Huynh *et al*. 2019; Hapsari *et al*. 2023). The negative allometric growth indicates that *N. marginatus* populations in the northern coastal waters of Central Java is categorised normal. However, signs of fishing pressure and benthic habitat degradation were also observed. Therefore, size-based fisheries management and restrictions on fishing effort should be imposed to ensure the sustainable growth of demersal fish stocks (Halim *et al*. 2020; Melnychuk *et al*. 2021).

The comparison between the length at first capture (Lc_50%_) and the length at first maturity (Lm_50%_) reflects population sustainability. Lc_50%_ that is smaller than Lm_50%_ implies that most individuals are caught before they have a chance to reproduce, which can reduce the stock’s regenerative capacity. The relatively small Lc_50%_ values observed in this study are likely associated with the use of small-mesh fishing gears such as mini trawls, *arad* and *cantrang*, as well as high fishing intensity in shallow coastal areas (Hartati *et al*. 2017; Sreekanth *et al*. 2017).

Different growth coefficients (*K*) and mortality rates reflect the variation in fishing pressure. Greater *K* value in Batang indicates faster growth but a shorter lifespan with greater exploitation intensity. High fishing intensity tends to cause populations to be dominated by smaller individuals (Pauly & Munro 1984; Amorim *et al*. 2020). Furthermore, the *E* value in Batang Regency reached 0.52 (over-exploitation) and reached *E* = 0.43 in Pati (within sustainable limits). Likewise, Hartuti *et al*. (2017) and Kindong *et al*. (2018) reported that *Nemipterus* spp. stocks in the Java Sea are under pressure from unselective demersal gears such as trawls.

The analysis of recruitment patterns also revealed distinctive dynamics, with peak in the first transitional season (March–July), when environmental conditions favour increased temperature and primary productivity (Camp *et al*. 2021). The differences in recruitment peaks between Pati and Batang are likely influenced by local variations such as water depth, substrate composition, and fishing intensity (Balachandran *et al*. 2024).

Overall, *N. marginatus* populations in Pati and Batang waters are reaching the threshold of sustainable fishing. Therefore, adaptive management strategies require biotechnical considerations, including the need to set minimum catch size in accordance with Lm, limit the fishing based on recruitment peaks (March–July and September), and set the ideal number of demersal fishing gears. The implementation of a Total Allowable Catch (TAC) near the Maximum Sustainable Yield (MSY) level is recommended to balance economic benefits with stock sustainability in the northern Java coastal region (Harford *et al*. 2018; Retnoningtyas *et al*. 2021; Osathanunkul & Suwannapoom 2023).

## CONCLUSION

The length-weight relationship of *N. marginatus* in Pati Regency and Batang Regency are both indicating a negative allometric growth pattern, with W = 0.134L^2.157^ and W = 0.052L^2.471^, respectively. The Lc_50%_ values for Pati and Batang were 11.61 cm and 10.90 cm, respectively. Growth parameter analysis showed that in Pati, *N. marginatus* had *L*∞ = 26.0 cm, *K* = 0.40 year^−1^, *M* = 1.02 year^−1^, *F* = 0.76 year^−1^, *Z* = 1.78 year^−1^, and *E* = 0.43, indicating a sustainably exploited condition. Whereas in Batang Regency, the *L*∞ = 26.0 cm, *K* = 0.58 year^−1^, *M* = 1.30 year^−1^, *F* = 1.40 year^−1^, *Z* = 2.70 year^−1^, and *E* = 0.52 (over-exploited). The estimated MSY was 3.286 tons (equivalent to IDR 29.574 million) in Pati and 3.589 tons (equivalent to IDR 32.302 million) in Batang. Recruitment peaks occur from February to July in Pati and from February to June, with additional peaks in September and November, in Batang. *N. marginatus* populations in the northern coastal waters of Central Java are approaching their optimum exploitation level. To ensure long-term sustainability, future studies should be conducted over longer periods (one year or more) and integrate both analytical and surplus production models. Biological and habitat-based research is also needed to determine key parameters such as Lm (length at first maturity), fishing grounds, spawning grounds, nursery grounds and migration routes.

## Figures and Tables

**FIGURE 1:**
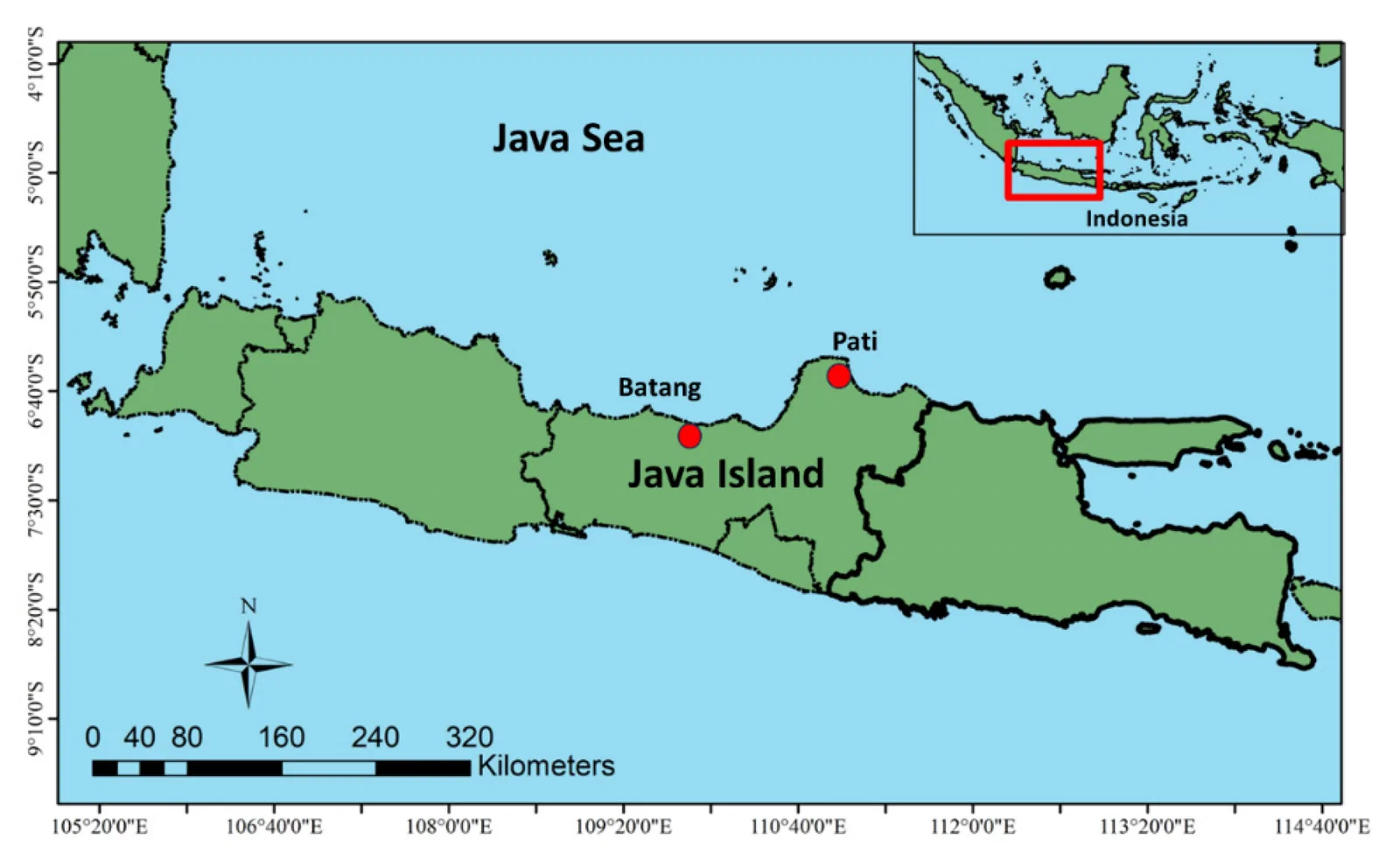
Research location (Source: Indonesia Information Geospatial Agency).

**FIGURE 2:**
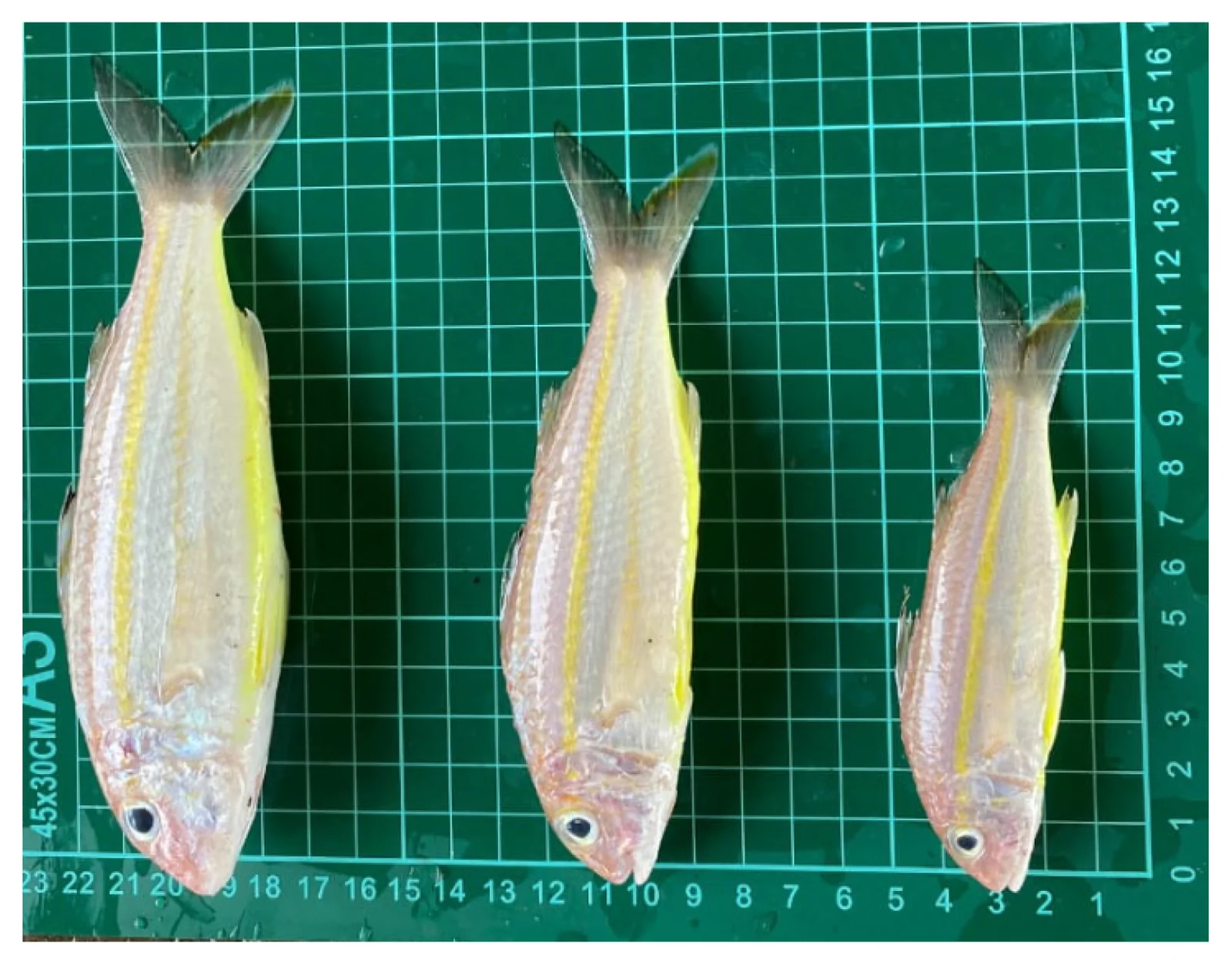
Specimen of *N. marginatus*.

**FIGURE 3:**
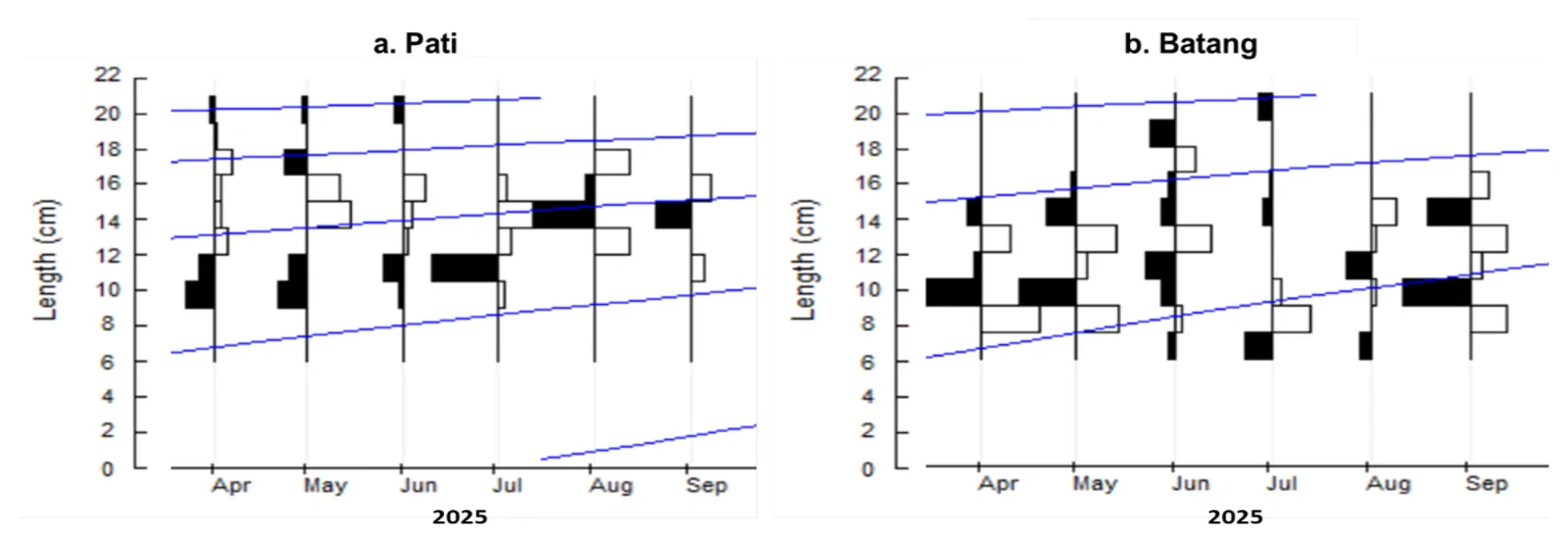
Size distribution of *N. marginatus*.

**FIGURE 4:**
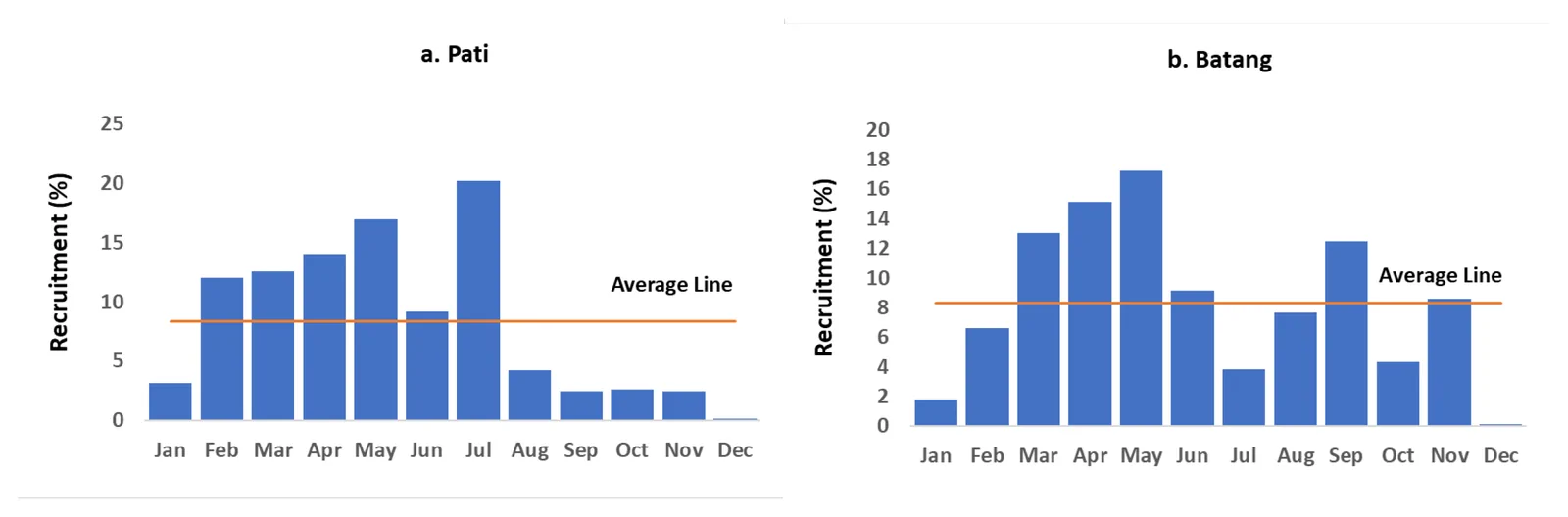
Recruitment pattern.

**TABLE 1:** Length-weight relationship of *Nemipterus* spp.

Species	Location	*a*	*b*	Growth pattern	References
*N. marginatus*	Pati, Indonesia	0.134	2.16	Negative allometric	This study
*N. marginatus*	Batang, Indonesia	0.052	2.47	Negative allometric	This study
*Nemipterus spp*.	Java Sea, Indonesia	0.00006	2.65	Negative allometric	Hartati *et al*. (2017)

**TABLE 2:** Size composition of *N. marginatus* caught in Pati and Batang.

Interval of TL (cm)	Middle value	Frequency		Percentage (%)		Cumulative percentage (%)	

		Pati	Batang	Pati	Batang	Pati	Batang
6.00–7.50	6.75	0	29	0	7	0	7
7.51–9.00	8.25	0	17	0	4	0	12
9.01–10.50	9.75	48	88	20	23	20	34
10.51–12.00	11.25	64	80	26	20	46	55
12.01–13.50	12.75	38	37	16	9	62	64
13.51–15.00	14.25	43	94	18	24	80	88
15.01–16.50	15.75	19	33	8	8	88	97
16.51–18.00	17.25	13	4	5	1	93	98
18.01–19.50	18.75	5	6	2	2	95	99
19.51–6.50	21.00	12	3	5	1	100	100

**TABLE 3:** Lc_50%_ vs. Lm_50%_ of *N. marginatus*.

Location	Lc_50%_	Reference
Pati Regency	11.61	This study
Batang Regency	10.90	This study
	Lm_50%_	
Tangerang, Banten	14.4	Hartati *et al*. (2017)

**TABLE 4:** Growth, mortality and exploitation parameters.

Location	L_∞_ (cm)	*K*	*M*	*F*	*Z*	*E*	Notes	References
Pati (*N. marginatus)*	26.0	0.40	1.02	0.76	1.78	0.43	Under-exploited	This study
Batang (*N. marginatus)*	26.0	0.58	1.30	1.40	1.02	0.52	Over-exploited	This study
Suez Gulf, Red Sea (*N. japonicus*)	31.66	0.24	0.61	1.14	1.74	0.65	Over-exploited	El-Ganainy *et al*. (2018)
Suez Gulf, Red Sea (*N. bipunctatus*)	29.55	0.26	0.67	1.05	1.72	0.61	Over-exploited	El-Ganainy *et al*. (2018)
Suez Gulf, Red Sea (*N. zysron*)	24.68	0.38	0.82	0.91	1.73	0.53	Over-exploited	El-Ganainy *et al*. (2018)
Java Sea (*Nemipterus* spp.)	25.8	0.45	0.90	0.84	1.74	0.48	Under-exploited	Hastuti *et al*. (2017)

**TABLE 5:** MSY estimation.

Location	Production assumption (ton)	Production estimation at MSY (ton)	Economic value at MSY (IDR)
Pati Regency	2.806	3.286	29.574 million
Batang Regency	3.722	3.589	32.302 million

*Note*: Economic valuation was calculated based on an assumed market price of IDR 9,000 per kilogram
